# *In Vivo* and *In Vitro* Antitumor Effects of Platycodin D, a Saponin Purified from Platycodi Radix on the H520 Lung Cancer Cell

**DOI:** 10.1155/2014/478653

**Published:** 2014-11-13

**Authors:** Jae Chan Park, Young Joon Lee, Hae Yun Choi, Yong Kook Shin, Jong Dae Kim, Sae Kwang Ku

**Affiliations:** ^1^Department of Pulmonary Internal Medicine of Anatomy and Histology, College of Korean Medicine, Daegu Haany University, No. 1 Haanydae-ro, Gyeongsan, Gyeongbuk 712-715, Republic of Korea; ^2^Department of Preventive Medicine, College of Korean Medicine, Daegu Haany University, Gyeongsan 712-715, Republic of Korea; ^3^The Medical Center for Globalization of Herbal Medicine, Daegu Haany University, Gyeongsan 712-715, Republic of Korea; ^4^Department of Natural Medicine Resources, Semyung University, Hecheon 390-711, Republic of Korea; ^5^Department of Anatomy and Histology, College of Korean Medicine, Daegu Haany University, No. 1 Haanydae-ro, Gyeongsan, Gyeongbuk 712-715, Republic of Korea

## Abstract

Platycodin D is a major pharmacological constituent of Platycodi radix and has showed various pharmacological activities through oxidative stress defense mechanisms. Here, possible antitumor, anticachexia, and immunomodulatory activities of platycodin D were observed on the H520 tumor cell-bearing athymic nude mice after confirming the *in vitro* cytotoxicity. Platycodin D was orally administered at dose levels of 200, 100, and 50 mg/kg, once a day for 35 days from 15 days after implantation. The results were compared with gemcitabine 160 mg/kg intraperitoneally treated mice (7-day intervals). Platycodin D showed favorable cytotoxic effects on the H520 cells, and also dose-dependently decreased the tumor volumes and weights with increases of apoptotic cells (caspase-3 and PARP immunopositive cells), iNOS and TNF-*α* immunoreactivities, decreases of COX-2 immunoreactivities in tumor masses. Platycodin D also showed dose-dependent immunostimulatory and anticachexia effects. Gemcitabine showed favorable cytotoxity against H520 tumor cell and related *in vivo* antitumor effects but aggravated the cancer related cachexia and immunosuppress in H520 tumor cell-bearing athymic nude mice. Taken together, it is considered that oral treatment of platycodin D has potent antitumor activities on H520 cells through direct cytotoxic effects, increases of apoptosis in tumor cells, and immunostimulatory effects and can be control cancer related cachexia.

## 1. Introduction

Lung cancer is currently the leading cause of cancer death worldwide [1], and surgical resection for cure is often only applicable to early-stage disease. The 5-year survival rate for all stages of lung cancer is only 15% [2]. Approximately 85% of lung cancer patients belong to the non-small-cell lung cancer (NSCLC) group with a poor prognosis [3]. Current treatment strategies for advanced lung cancer include surgical resection, radiation, cytotoxic chemotherapy, and, more recently, photodynamic therapy [4]. Although a combination of chemotherapy and radiation can improve survival, most patients die of disease progression, often resulting from acquired or intrinsic resistance to chemotherapeutic drugs [5]. In addition, in almost two thirds of cases, the cancer has already spread beyond localized disease at the time of diagnosis [6, 7], limiting the options for therapy. Cachexia is characterized by major metabolic abnormalities and maladaptations. Often food/energy intake is reduced, resting energy expenditure is increased, and catabolism is accelerated [8]. Cachexia is associated with anorexia, fat- and muscle-tissue wasting, and a progressive deterioration in the quality of life [9]. As many as 80% of all patients with cancer develop cachexia before death, and, in over 20% of these patients, cachexia is the primary cause of death [10, 11]. T-cell mediated immunosuppress are directly related to cancer occurrences or in cancer patient, marked immune depresses are also observed [12, 13], and recently, stimulation or increases of body immune systems are highlighted as a new treatment regimens for cancer therapy [14, 15].

Natural herbs contain various phenolic compounds, vitamins, carotenoids, and flavonoids, and they have showed various pharmacological effects including ant-oxidative, antiallergic, and anticancer effects [16]. Platycodi Radix, the roots of* Platycodon grandiflorum* (Jacq.), have been used traditionally as an expectorant and a remedy for bronchitis, tonsillitis, laryngitis, and suppurative dermatitis in China, Korea, and Japan. In China and Korea, the fresh roots of* P. grandiflorum* have been eaten as pickles for preventing obesity [17]. Platycodin D is a major pharmacological constituent of Platycodi radix [18] and has been showed favorable antioxidant effect mediated antidiabetic [19], anti-inflammatory [20], antinociceptive [21], and immunomodulatory [22] activities. Particularly, platycodin D showed favorable antitumor effects against A549 cells and adenocarcinomic human alveolar basal epithelial cells [23, 24], but the anticancer effects on H520 cells, a representative NSCLC cell lines of platycodin D, were not revealed upon our knowledge. In this study, the cytotoxicity of platycodin D was evaluated in* in vitro* experiment, and then, potential antitumor activities were observed using H520 tumor cell bearing athymic nude mice after 35 days continuous oral treatment. The cytotoxicity was tested by MTT assay against H520 cells, and the antitumor, anticachexia and immunomodulatory effects were observed in H520 cell xenograft Balb/c* nu*-*nu* nude mice. The results were compared with gemcitabine 160 mg/kg, 7-day intervals, intraperitoneal treated mice, as a reference drug [25, 26] in this experiment.

## 2. Materials and Methods

### 2.1. Preparations of Test Materials

**Figure 1:**
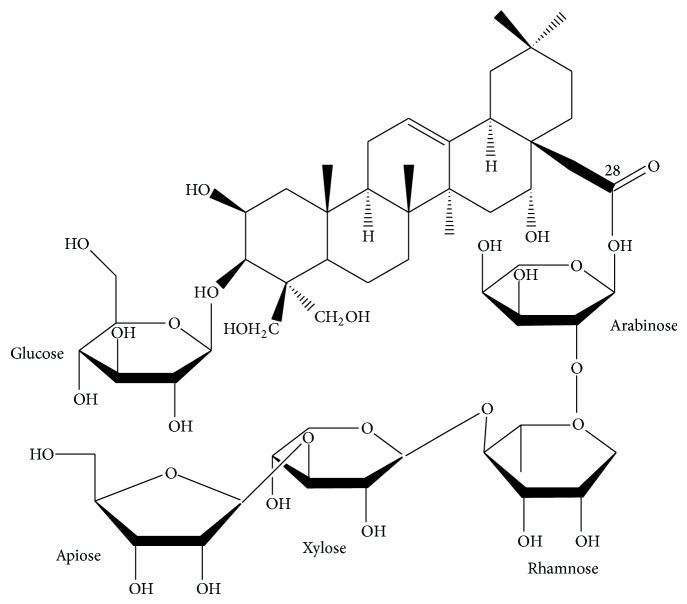
Chemical structure of platycodin D, a triterpenoid bidesmoside, composed of an aglycone moiety, 3-Glc and 28-O-Api-Xyl-Rha-Ara, used in this study.

The platycodin D, gift from Glucan Corp., Ltd. (Pusan, Korea), was extracted from Platycodi radix by previous method [19]. The raw sample (100 kg) of Platycodi radix was extracted with methanol and partitioned sequentially with n-hexane, chloroform, ethyl acetate, and n-butanol. The n-butanol fraction was then subjected to Diaion HP-20 resin (Mitsubishi, Tokyo, Japan), and the fractions eluted at 60–80% of methanol were collected to obtain 90 g of crude saponins. The crude saponins were further purified by repeated silica gel (Merck, Darmstadt, Germany) chromatography to obtain the purified platycodin D. The yield of purified platycodin D was 2% of crude saponin. The process was repeated several times until a sufficient quantity of platycodin D was obtained. The purified platycodin D was identified on the basis of Rf, FAB-MS (=1225.38), and [13C]-NMR spectra compared with the authentic platycodin D (Figure 1). Light yellow powders of platycodin D were used as test material, and white crystalline powders of gemcitabine (Sigma-Aldrich, St. Louise, MO, USA) were used as control reference drug.

### 2.2. *In Vitro* MTT Assay

H520 (human NSCLC, non-small-cell lung squamous cell carcinoma; American Type Culture Collection Center, VA, USA) cells were maintained in RPMI 1640 media (Life Technologies, Grand Island, NY, USA) containing 10% fetal bovine serum (FBS; Life Technologies, Grand Island, NY, USA), 100 U/mL penicillin (Sigma-Aldrich, St. Louise, MO, USA), and 100 *μ*g/mL streptomycin (Sigma-Aldrich, St. Louise, MO, USA) at 37°C and 5% CO_2_ conditions. MTT (Sigma-Aldrich, St. Louise, MO, USA) assay was performed to determine cell proliferation, the cytotoxic effects of test materials. Briefly, H520 cells were plated in 96-well plates at a density of 1 × 10^4^ cells/well. After incubating for 24 hrs, cells were treated with seven different concentrations of platycodin D (0, 1.56, 3.13, 6.25, 12.5, 25, and 50 *μ*g/mL in DMSO) or gemcitabine (0, 50, 100, 200, 400, 800, and 1600 *μ*g/mL) for 72 hrs. MTT 0.5 mg/mL solution in the culture medium was then added to the wells. After further 4 hrs of incubation, the medium was removed and DMSO was added to the plates. The optical density was measured at 570 nm using a microplate reader (Tecan; Männedorf, Switzerland) and then relative of negative control (0 *μ*g/mL) were calculated and IC_50_. Six independent MTT assays were repeatedly conducted in this experiment.

### 2.3. *In Vivo* H520 Cell Xenograft Mouse

#### 2.3.1. Animals and Husbandry

A total of 60 virgin, specific pathogen-free female Balb/c Slc-*nu/nu* mice (5-wk old upon receipt; Shizuoka Laboratory Animal Center, Shizuoka, Japan) were used after acclimatization for 42 days. Animals were allocated four to five per polycarbonate cage in a temperature (20–25°C) and humidity (45–55%) controlled room. Light: dark cycle was 12 hr : 12 hr and food (Samyang, Seoul, Korea) and water were supplied free to access. Fifty mice were used as tumor-bearing mice and remainder ten mice were used as intact control in this experiment. Fourteen days after tumor inoculation, eight mice per group showing regular tumor volumes (averages, about 130 mm^3^; individual ranges, 101.79~162.78 mm^3^) and body weights (averages, about 21 g; individual ranges, 17.90~24.80 g) were selected including eight intact control mice showed regular body weights (averages, about 22 g; individual ranges, 20.70~24.20 g) and used further experiments in the present study. Animal experiments were conducted according to the national regulations of the usage and welfare of laboratory animals and approved by the Institutional Animal Care and Use Committee in Daegu Haany University (Gyeongsan, Gyeongbuk, Korea) (Approval number 2013-023).

#### 2.3.2. Tumor Cells Xenograft and Drug Administration

Maintained H520 cells were suspended in saline as 1 × 10^8^ cell/mL concentration, and 0.2 mL of cell suspensions (2 × 10^7^ cell/mouse) were subcutaneously inoculated on the right dorsal hip skins of each mouse, and equal volume of saline was also subcutaneously injected in intact control mice, instead of tumor cell suspensions. Three different dosages of Platycodin D (200, 100, and 50 mg/kg) were orally administered, once a day for 35 days from 15 days after tumor cell inoculations (reached tumor volumes as 100 mm^3^, at least) in a volume of 10 mL/kg (of body weight). Gemcitabine was dissolved in saline and intraperitoneally administered from 15 days after tumor cell inoculation, 7-day intervals in a volume of 10 mL/kg. In intact and vehicle controls, only distilled water 10 mL/kg was orally administered, instead of Platycodin D or gemcitabine. The dosage of platycodin D—200, 100 or 50 mg/kg was selected based on the previous animal efficacy tests [27, 28]. In addition, the dosage of gemcitabine, 160 mg/kg, 7-day intervals, and intraperitoneal treatment was also selected based on the previous efficacy tests in H520 cell xenograft nude mice [25, 26].

#### 2.3.3. Body Weight Measurements

Changes of body weight were measured at 1 day before administration (14 days after tumor cell inoculation), initiation of administration, 1, 7, 14, 21, 28, and 34 days after initiation of administration with sacrifice using an automatic electronic balance (Precisa Instrument, Dietikon, Switzland), respectively. At start of administration and at a termination, all animals were fasted overnight (water was not; about 18 hrs) to reduce the differences from feeding.

#### 2.3.4. Tumor Volume Measurements

Tumor length (long axis) and tumor width (short axis) of each tumor-bearing mouse, not in intact control mice were measured at 1 day before administration (14 days after tumor cell inoculation), initiation of administration, 1, 3, 7, 14, 21, 28, 34, and 35 days after initial administration using electronic digital caliper (Mytutoyo, Tokyo, Japan), and tumor volumes were calculated as 1/2 × length × width^2^ (mm^3^) according to previous method [29].

#### 2.3.5. Tumor Weight Measurements

At sacrifice, tumor masses in each mouse were collected after eliminations of the surrounding skins, connective tissues, muscles, and any debris. The weight of tumor was measured at g levels regarding absolute wet-weights. To reduce the individual body weight differences, the relative weight (%) was calculated using body weight at sacrifice and absolute tumor weights as (absolute tumor weights/body weight at sacrifice) × 100.

#### 2.3.6. Lymphatic and Periovarian Fat Pad Weight Measurements

At sacrifice, spleen, left submandibular lymph node, and left periovarian fat pads in each mouse were collected after eliminations of the surrounding connective tissues, muscles, and any debris. The weight of organs was measured at g levels regarding absolute wet-weights. To reduce the individual body weight differences, the relative weight (%) was also calculated using body weight at sacrifice and absolute organ weights as (absolute organ weights/body weight at sacrifice) × 100.

#### 2.3.7. Serum Interleukin (IL)-6 and Interferon (IFN)-*γ* Level Measurements

For serum biochemistry, 1 mL of whole blood was collected from vena cava at sacrifice under Zoletile mixture (Zoletile 50; Virbac Lab., Paris, France) anesthesia and separated the serum. All serum samples were frozen at −150°C until they were assayed. Serum IL-6 levels werosteocalcin levels were detected by enzyme-linked immunosorbent assay (ELISA) kit (R&D Systems Inc., Minneapolis, MN, USA) as pg/mL according to previously established method [30], and serum IFN-*γ* levels were also calculated using mouse IFN-*γ* ELISA kit (BD Biosciences/Pharmingen, San Diego, CA, USA) according to manufacturer's recommended protocols as pg/mL levels.

#### 2.3.8. Natural Killer (NK) Cell Activity Measurements

Splenic and peritoneal NK cell activities were measured by the use of a standard ^51^Cr release assay [31]. Briefly, all mice were killed at sacrifice, and splenocytes and peritoneal macrophages were collected from each mouse. Spleen 10~20 mg were separated and washed by RPMI-1640 medium (Life Technologies, Grand Island, NY, USA), twice at 4°C and homogenates were prepared, and peritoneal macrophages were collected by repeat intraperitoneally wash of RPMI medium and then resuspended. Prepared splenic and peritoneal NK cells disrupted mechanically by maceration through a wire mesh (Mesh number 100, Sigma-Aldrich, St. Louise, MO, USA) wetted with RPMI-1640 medium. The mesh was washed with RPMI-1640 medium to collect as many cells as possible. The debris was allowed to settle, and the cell suspension was pelleted by centrifugation. RBC was lysed by resuspending the pellet in cold 1% ammonium oxalate and incubating on ice for 10 min. The cells were pelleted and washed twice with HBSS (Hanks Balanced Salt Solution; Life Technologies, Grand Island, NY, USA). The peritoneal macrophages (1 × 10^5^ cells/mL~2 × 10^5^ cells/mL) were cultured overnight in complete Medium (Sigma-Aldrich, St. Louise, MO, USA). Splenocytes were cultured overnight in Dulbecco's Modified Eagle Medium (Invitrogen, NY, USA) in the absence or presence of recombinant IL-2 (1000 IU/mL; Proleukin Chiron, Emeryville, CA, USA). The HTLA-230 neuroblastoma target cells were labeled for 2 hrs with Na_2_^51^CrO_4_ (100 *μ*Ci/1 × 10^6^ cells) (ICN Biomedicals, Asse, Belgium). Target cells were then incubated for 6 hrs at 37°C with splenocytes or peritoneal macrophages as effector cells. The effector: target cell ratio was 100 : 1 for splenocytes and 10 : 1 for macrophages. Supernatants were collected, and the amount of radioactivity released into the supernatants was counted with a gamma counter (Cobra 5002; Canberra Packard, Meriden, CT, USA). The percentage of specific target cell lysis was calculated as [(Exp − *S*)/(*M* − *S*) × 100]% (where Exp is the observed released ^51^Cr value, *S* is the spontaneously released ^51^Cr value, and *M* is the maximum released ^51^Cr value).

#### 2.3.9. Splenic Cytokine Content Measurements

Splenic concentrations of tumor necrosis factor (TNF)-*α*, IL-1*β*, and IL-10 were measured by ELISA using commercially available kits, mouse TNF-*α* ELISA kit (BD Biosciences/Pharmingen, San Diego, CA, USA), mouse IL-1*β* ELISA kit (Genzyme, Cambridge, MA, USA) and mouse IL-10 ELISA kit (Genzyme, Cambridge, MA, USA), respectively, as previously described [32]. Approximately 10–15 mg of tissue samples was homogenized in a tissue grinder containing 1 mL of lysis buffer (PBS containing 2 mM PMSF and 1 mg/mL of aprotinin, leupeptin, and pepstatin A) as described by Clark et al. [33]. Analysis was performed with 100 mL of standard (diluted in lysis buffer) or 10, 50, or 100 mL of tissue homogenate. Each sample was run in duplicate, and a portion of the sample was analyzed for protein. Data are expressed as pg/mg of protein. For each assay a standard curve was generated and, based on replicates of the measured absorbance, demonstrated an average coefficient of variance of <10%.

#### 2.3.10. Histopathology

After weight measurement at sacrifice, some parts of tumor mass, spleen, left side of submandibular lymph node and left periovarian fat pads were separated and fixed in 10% neutral buffered formalin, at least 24 hrs. Then paraffin-embedded and 3 *μ*m-thick sections were prepared. Each slide was then stained with Hematoxylin and eosin for general histopathology. Histological evaluation was performed on the central zone of the each organ or tumor mass, whenever possible. The histopathologist was blinds to group distribution when this analysis was made. To observe more detail changes, the tumor cell volumes, intact tumor cell occupied regions (%/mm^2^ of tumor mass), were calculated in each prepared tumor mass histological specimens using automated image analyzer (iSolution FL ver 9.1, IMT i-solution Inc., Quebec, Canada) under microscopy (Nikon, Tokyo, Japan). Total thickness of central cross trimmed spleen (from apex of anterior border to centre of posterior border; mm/spleen), white pulp numbers (/mm^2^ of spleen) and diameters (*μ*m/white pulps), total submandibular lymph node thicknesses (*μ*m/central regions), number of cortex lymphoid follicles (numbers/mm^2^ of cortex) and cortex thicknesses (*μ*m/lymph node) were also calculated according to previous report [32]. In addition, total thicknesses (*μ*m/central regions), and mean diameters of white adipocyte (*μ*m/white adipocytes) were measured by automated image analyzer according to previous established method [34].

#### 2.3.11. Immunohistochemistry

After deparraffinized of prepared tumor mass histological paraffin sections, citrate buffer antigen (epitope) retrieval pretreatment were conducted as previously [35]. Briefly, preheat water bath with staining dish containing 10 mM citrate buffers (pH 6.0) until temperature reaches 95–100°C. Immerse slides in the staining dish and place the lid loosely on the staining dish. Incubate for 20 minutes and turn off the water bath. Place the staining dish at room temperature and allow the slides to cool for 20 minutes. After epitope retrievals, sections were immunostained using avidin-biotin complex (ABC) methods for caspase-3-, cleaved poly (ADP-ribose) polymerase (PARP), cyclooxygenase-2 (COX-2), inducible nitric oxide synthases (iNOS) and TNF-*α* [36, 37]. Briefly, endogenous peroxidase activity was blocked by incubated in methanol and 0.3% H_2_O_2_ for 30 minutes, and nonspecific binding of immunoglobulin was blocked with normal horse serum blocking solution (Vector Lab., Burlingame, CA, USA. Dilution 1 : 100) for 1 hr in humidity chamber. Primary antiserum (Table 1) were treated for overnight at 4°C in humidity chamber, and then incubated with biotinylated universal secondary antibody (Vector Lab., Burlingame, CA, USA. Dilution 1 : 50) and ABC reagents (Vectastain Elite ABC Kit, Vector Lab., Burlingame, CA, USA; Dilution 1 : 50) for 1 hr at room temperature in humidity chamber. Finally, the section was reacted with peroxidase substrate kin (Vector Lab., Burlingame, CA, USA) for 3 min at room temperature. All sections were rinse in 0.01 M PBS for 3 times, between steps. The cells showed stronger immunoreactivities in the cytoplasm, over 20%, the density, against each antiserum were regarded as positive. The percentages regions occupied by caspase-3-, PARP-, COX-2-, iNOS-, and TNF-*α*-positive cells located in tumor mass were measured by automated image analyzer (%/mm^2^ of tumor mass), respectively.

### 2.4. Statistical Analyses

Numerical data are presented as means ± SDs, and multiple comparison tests for the different dose groups were conducted. Homogeneity of variance was examined using the Levene test. If the Levene test indicated no significant deviations from homogeneity in the variance, the data were analyzed by one-way analysis of variance followed by Scheffe's test to determine the group comparisons that were significantly different. In case of significant deviations from variance homogeneity was observed at Levene test, a nonparametric comparison test, Kruskal-Wallis H test was conducted. When a significant difference is observed in the Kruskal-Wallis H test, the Mann-Whitney *U* (MW) test was conducted to determine the specific pairs of group comparison, which are significantly different. Statistical analyses were conducted using SPSS for Windows (Release 14.0 K, IBM SPSS Inc., Armonk, NY, USA), and *P* values < 0.05 were considered significantly different.

## 3. Results

### 3.1. Results on the MTT Assay

**Figure 2:**
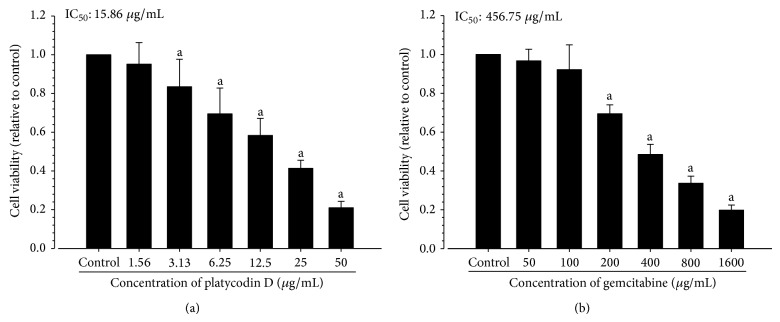
Effects of platycodin D (a) and gemcitabine (b) on the H520 tumor cell viabilities by MTT assay. Values are expressed as Mean ± SD of six independent experiments; ^a^ *P* < 0.01 as compared with control (0 *μ*g/ml) by MW test.

Significant (*P* < 0.01) decreases of survivability of H520 tumor cells were demonstrated in platycodin D treatment from 3.13 *μ*g/mL to 50 *μ*g/mL concentration, and therefore, the IC_50_ of platycodin D against H520 tumor cells was calculated as 15.86 *μ*g/mL in this study (Figure 2). In addition, significant (*P* < 0.01) decreases of survivability of H520 tumor cells were also noticed from 200 *μ*g/mL to 1600 *μ*g/mL of gemcitabine, and therefore, the IC_50_ of gemcitabine against H520 tumor cells was calculated as 456.75 *μ*g/mL (1.74 *μ*M) in this experiment (Figure 2).

### 3.2. Results on the H520 Cell Xenograft Nude Mice

#### 3.2.1. Effects on the Body Weights

**Figure 3:**
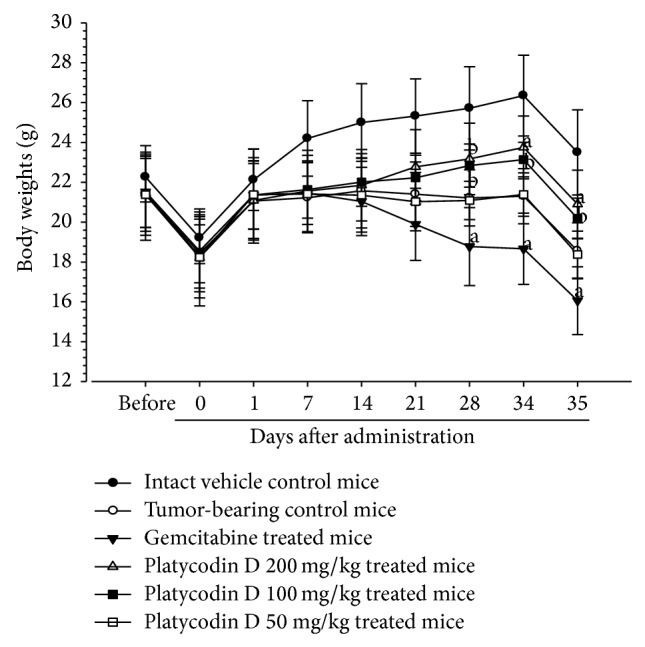
Body weight changes in h520 tumor cell xenograft mice. Values are expressed as Mean ± SD of eight mice (g). All animals at sacrifice and Day 0 (initiation day of treatment) were overnight fasted; gemcitabine was intraperitoneally administered at 160 mg/kg, 7-day intervals; platycodin D was orally administered, once a day; ^a^ *P* < 0.01 and ^b^ *P* < 0.05 as compared with tumor-bearing control by LSD test.

Significant (*P* < 0.01) decreases of the body weights were demonstrated in all tumor-bearing mice as compared with intact control from 7 days after initial administration. Gemcitabine 160 mg/kg, 7-day intervals, intraperitoneally treated mice showed significant decreases of body weights as compared with tumor-bearing control mice from 28 days after initial administration. However, platycodin D 200 and 100 mg/kg treatment significantly (*P* < 0.01 or *P* < 0.05) increased the body weights as compared with tumor-bearing control mice from 28 days after administration, but platycodin D 50 mg/kg did not induce any meaningful body weight changes (Figure 3).

#### 3.2.2. Effects on the Tumor Volumes

**Figure 4:**
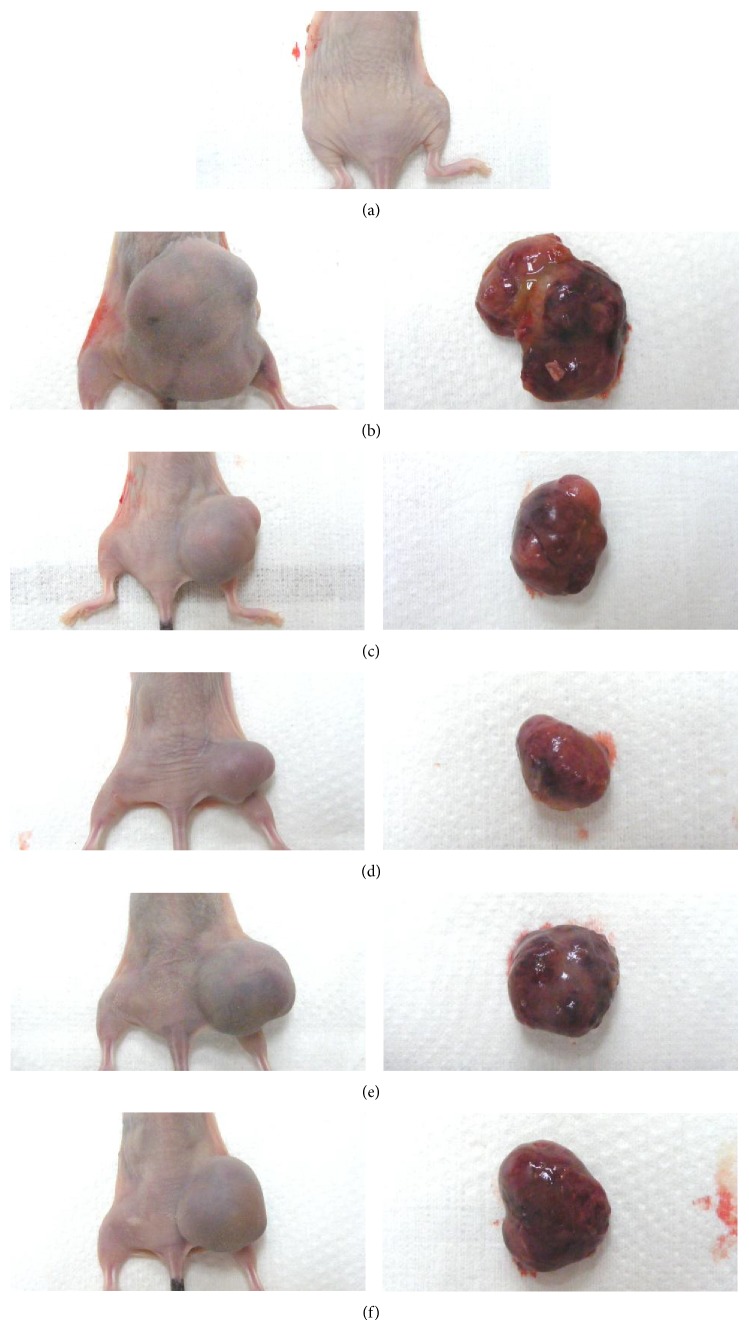
Representative gross images of dorsal back regions taken from intact vehicle control (a) or tumor masses taken from tumor-bearing control (b), gemcitabine (c), platycodin D 200 (d), 100 (e), and 50 (f) mg/kg treated mice. Gemcitabine was intraperitoneally administered at 160 mg/kg, 7-day intervals; platycodin D was orally administered, once a day.

**Figure 5:**
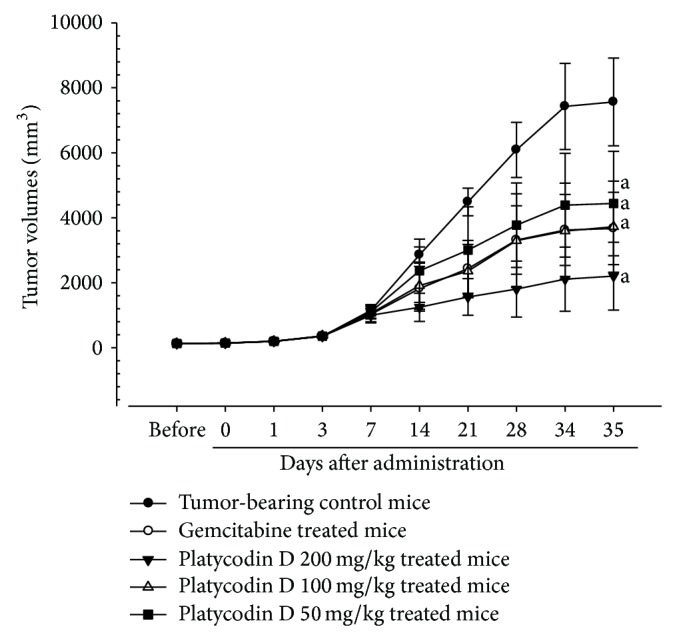
Tumor volume changes in H520 tumor cell xenograft mice. Values are expressed Mean ± SD of eight mice (mm^3^). Gemcitabine was intraperitoneally administered at 160 mg/kg, 7-day intervals; platycodin D was orally administered, once a day; ^a^ *P* < 0.01 as compared with tumor-bearing control by LSD test.

Significant (*P* < 0.01) decreases of the tumor volumes were detected in gemcitabine treated mice as compared with tumor-bearing control from 14 days of administration in this experiment. Platycodin D 200 and 100 mg/kg treated mice also showed significant (*P* < 0.01 or *P* < 0.05) decreases of tumor volumes from 14 days after initial administration, and platycodin D 50 mg/kg administered mice also showed significant (*P* < 0.01 or *P* < 0.05) decreases of tumor volumes from 21 days of administration, respectively (Figures 4 and 5).

#### 3.2.3. Effects on the Tumor Weights

Significant (*P* < 0.01 or *P* < 0.05) decreases of the tumor absolute and relative (% of body weight) weights were observed in all drug administered mice including platycodin D 50 mg/kg treated mice as compared with tumor-bearing control at sacrifice. Platycodin D showed obvious dose-dependent decreases of tumor weights at sacrifice as compared with tumor-bearing control mice, respectively (Table 2, Figure 4).

#### 3.2.4. Effects on the Spleen Weights

Significant (*P* < 0.01 or *P* < 0.05) decreases of the spleen absolute and relative weights were observed in tumor-bearing control mice as compared with intact control mice, respectively. However, all three different dosages of platycodin D administered mice showed significant (*P* < 0.01 or *P* < 0.05) increases of the spleen weights as compared with tumor-bearing control, dose-dependently. Gemcitabine 160 mg/kg treated mice showed significant (*P* < 0.05) decreases of the absolute weight but quite similar relative spleen weights as compared with tumor-bearing control mice, respectively (Table 2).

#### 3.2.5. Effects on the Submandibular Lymph Node Weights

Significant (*P* < 0.01) decreases of the submandibular lymph node absolute and relative weights were observed in tumor-bearing control mice as compared with intact control mice, respectively. However, all three different dosages of platycodin D administered mice showed significant (*P* < 0.01) increases of submandibular lymph node weights as compared with tumor-bearing control, dose-dependently. Gemcitabine 160 mg/kg treated mice showed marked decreases of the absolute submandibular lymph node weight but quite similar relative submandibular lymph node weights as compared with tumor-bearing control mice, respectively (Table 2).

#### 3.2.6. Effects on Periovarian Fat Pad Weights

Significant (*P* < 0.01) decreases of the periovarian fat pad absolute and relative weights were observed in tumor-bearing control mice as compared with intact control mice, respectively. However, all three different dosages of platycodin D administered mice showed significant (*P* < 0.01) increases of the periovarian fat pad weights as compared with tumor-bearing control, dose-dependently. Gemcitabine 160 mg/kg treated mice showed significant (*P* < 0.01 or *P* < 0.05) decreases of the absolute and relative periovarian fat pad weights as compared with tumor-bearing control mice, respectively (Table 2).

#### 3.2.7. Effects on the Serum IL-6 and IFN-*γ* Levels

**Figure 6:**
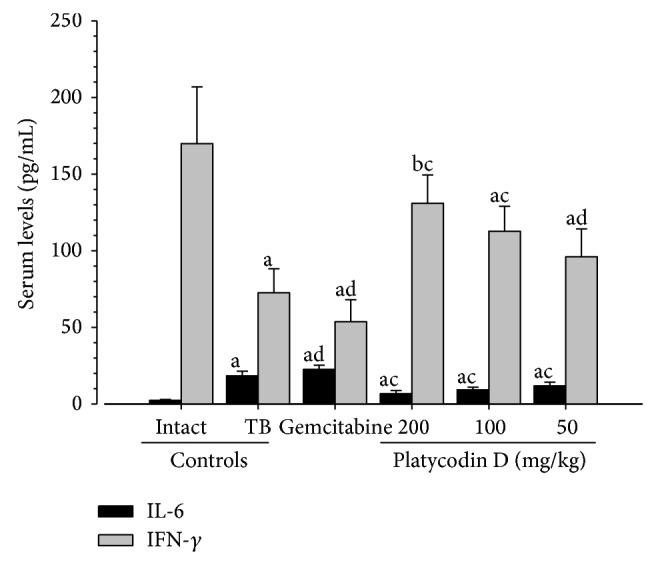
Changes on the serum IL-6 and IFN-*γ* levels in H520 tumor cell xenograft mice. Values are expressed Mean ± SD of eight mice (pg/ml); TB = tumor-bearing; IL = interleukin; IFN = interferon Gemcitabine was intraperitoneally administered at 160 mg/kg, 7-day intervals; Platycodin D was orally administered, once a day; ^a^ *P* < 0.01 and ^b^ *P* < 0.05 as compared with intact control by MW test; ^c^ *P* < 0.01 and ^d^ *P* < 0.05 as compared with TB control by MW test.

Significant (*P* < 0.01) increases of the serum IL-6 levels, and decreases of IFN-*γ* levels were observed in tumor-bearing control mice as compared with intact control mice, respectively. However, platycodin D 200, 100 and 50 mg/kg administered mice showed significant (*P* < 0.01 or *P* < 0.05) decreases of serum IL-6 levels, and increases of IFN-*γ* levels as compared with tumor-bearing control, dose-dependently. Gemcitabine 160 mg/kg treated mice showed significant (*P* < 0.05) aggravated the serum IL-6 and IFN-*γ* level changes, induced by H520 tumor-bearing in this study (Figure 6).

#### 3.2.8. Effects on the NK Cell Activity

**Figure 7:**
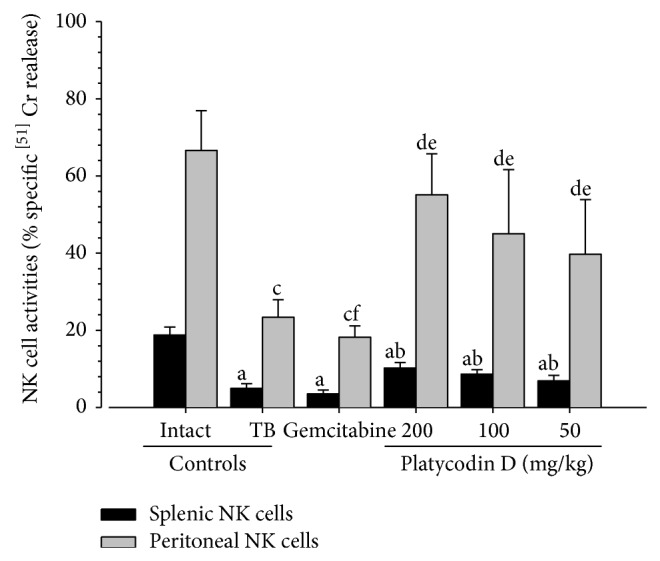
Changes on the splenic and peritoneal NK cell activities in H520 tumor cell xenograft mice. Values are expressed Mean ± SD of eight mice (% specific ^51^Cr releases); TB = tumor-bearing; NK = natural killer; Gemcitabine was intraperitoneally administered at 160 mg/kg, 7-day intervals; Platycodin D was orally administered, once a day; ^a^ *P* < 0.01 as compared with intact control by LSD test; ^b^ *P* < 0.01 as compared with TB control by LSD test; ^c^ *P* < 0.01 and ^d^ *P* < 0.05 as compared with intact control by MW test; ^e^ *P* < 0.01 and ^f^ *P* < 0.05 as compared with TB control by MW test.

Significant (*P* < 0.01) decreases of the splenic and peritoneal NK cell activities were observed in tumor-bearing control mice as compared with intact control mice, respectively. However, all three different dosages of platycodin D administered mice showed significant (*P* < 0.01) increases of the NK cell activities as compared with tumor-bearing control, dose-dependently. Gemcitabine administered mice showed marked decreases of the splenic NK cell activities and significant (*P* < 0.05) decreases of the peritoneal NK cell activities as compared with tumor-bearing control mice, respectively (Figure 7).

#### 3.2.9. Effects on the Splenic Cytokine Contents

Significant (*P* < 0.01) decreases of the splenic TNF-*α*, IL-1*β* and IL-10 contents were observed in tumor-bearing control mice as compared with intact control mice, respectively. However, platycodin D 200, 100 and 50 mg/kg administered mice showed significant (*P* < 0.01 or *P* < 0.05) increases of the splenic cytokine contents as compared with tumor-bearing control, dose-dependently. On the contrary, gemcitabine administered mice showed significant (*P* < 0.05) decreases of the splenic TNF-*α* and IL-1*β*, and nonsignificant but dramatic decreases of splenic IL-10 contents as compared with tumor-bearing control mice, respectively (Table 3).

#### 3.2.10. Effects on the Tumor Mass Histopathology and Immunohistochemistry

**Figure 8:**
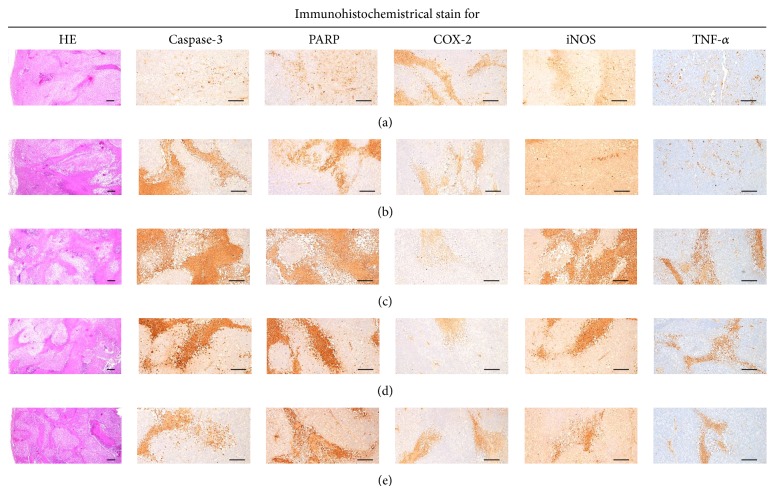
Representative histological images of tumor masses, taken from tumor-bearing control (a), gemcitabine (b), platycodin D 200 (c), 100 (d) and 50 (e) mg/kg treated mice. Gemcitabine was intraperitoneally administered at 160 mg/kg, 7-day intervals; Platycodin D was orally administered, once a day; HE = hematoxylin-eosin stain; PARP = cleaved poly(ADP-ribose) polymerase; COX-2 = cyclooxygenase-2; iNOS = inducible nitric oxide synthases; TNF = tumor necrosis factor; Scale bars = 167 *μ*m.

Relatively well-differentiated tumor cells, as squamous carcinoma were existed in the tumor mass of tumor-bearing control mice. However, the tumor cell volumes in the tumor masses were significantly (*P* < 0.01) decreased in all administered mice as compared with tumor-bearing control mice, respectively. Especially, platycodin D 200, 100 and 50 mg/kg treated mice showed obvious dose-dependent decreases of the tumor cell volumes (Table 4, Figure 8). In addition, all drug treated mice including platycodin D 50 mg/kg showed significant (*P* < 0.01 or *P* < 0.05) increases of caspase-3 and PARP immunoreactivities in the tumor mass and decreases of COX-2-immunolabeled cells as compared with tumor-bearing control mice, respectively (Table 4, Figure 8). Marked and dose-dependent increases of iNOS and TNF-*α* immunoreactivities were demonstrated in all platycodin D treated mice as compared with tumor-bearing mice, but gemcitabine 160 mg/kg did not influenced on the iNOS and TNF-*α* immunoreactivities in tumor masses (Table 4, Figure 8).

#### 3.2.11. Effects on the Histopathology of the Spleen

**Figure 9:**
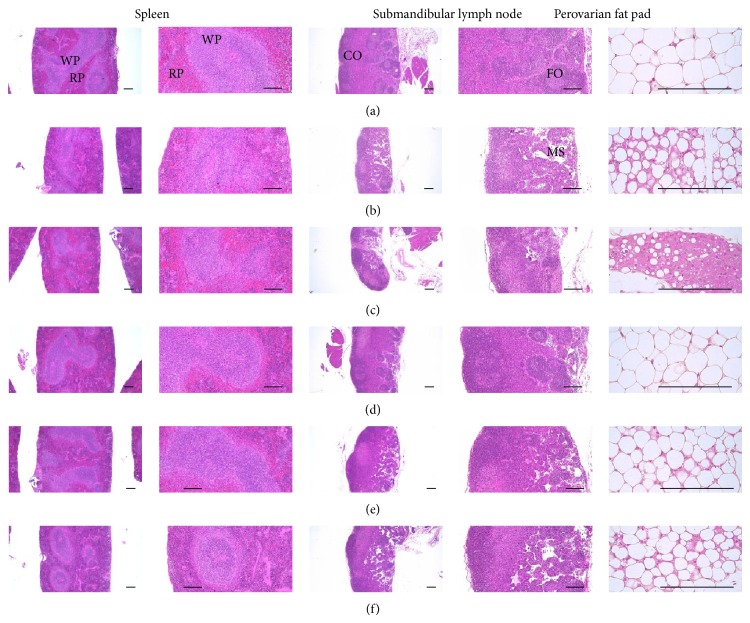
Representative histological images of spleen, submandibular lymph node and periovarian fat pad, taken from intact vehicle control (a), tumor-bearing control (b), gemcitabine (c), platycodin D 200 (d), 100 (e) and 50 (f) mg/kg treated mice. Gemcitabine was intraperitoneally administered at 160 mg/kg, 7-day intervals; Platycodin D was orally administered, once a day; All hematoxylin-eosin stain; WP = white pulp; RP = red pulp; FO = follicle; CO = cortex; MS = medullary sinus; Scale bars = 167 *μ*m.

Atrophic changes related to the decrease of the splenic white pulp lymphoid cells were detected in tumor-bearing control as compared with intact control; consequently the total splenic thicknesses, white pulp numbers and diameters were significantly (*P* < 0.01) decreased in tumor-bearing mice as compared with intact control, respectively. However, these splenic atrophic changes were significantly (*P* < 0.01 or *P* < 0.05) inhibited by treatment of platycodin D 200, 100 and 50 mg/kg, dose-dependently as compared with tumor-bearing control, respectively. Gemcitabine treated mice did not showed any significant changes on the total splenic thicknesses, white pulp numbers and diameters as compared with tumor-bearing control mice, respectively (Table 5, Figure 9).

#### 3.2.12. Effects on the Submandibular Lymph Node Histopathology

Marked atrophic changes related to the decrease of lymphoid cells were detected in the submandibular lymph nodes of tumor-bearing control as compared with intact control; consequently the total and cortex thicknesses and follicle numbers were significantly (*P* < 0.01) decreased in tumor-bearing control as compared with intact control, respectively. However, these submandibular lymph node atrophic changes were significantly (*P* < 0.01 or *P* < 0.05) inhibited by treatment of platycodin D 200, 100 and 50 mg/kg, dose-dependently as compared with tumor-bearing control, respectively. Gemcitabine treated mice did not showed any significant changes on the submandibular lymph node total and cortex thicknesses and follicle numbers as compared with tumor-bearing control mice, respectively (Table 5, Figure 9).

#### 3.2.13. Effects on the Periovarian Fat Pad Histopathology

Noticeable atrophic changes related to the decreases of the sizes of white adipose cells were detected in the periovarian fat tissues of tumor-bearing control as compared with intact control; consequently the total deposited fat thicknesses and mean diameters of white adipocyte were significantly (*P* < 0.05) decreased in tumor-bearing control as compared with intact control, respectively. However, these atrophic changes on the white periovarian adipose tissues were significantly (*P* < 0.01) inhibited by treatment of all three different dosages of platycodin D, dose-dependently, as compared with tumor-bearing control in this study. Gemcitabine administered mice showed significant (*P* < 0.01 or *P* < 0.05) decreases of the total deposited periovarian fat thicknesses and mean diameters of white as compared with tumor-bearing control mice, respectively (Table 5, Figure 9).

## 4. Discussion

Lung cancer develops in more than 200,000 people and causes more than 160,000 deaths each year; NSCLC is the most common type of lung cancer [38]. Many patients receive chemotherapy or radiation before surgical resection to shrink the tumor and may continue to receive treatment following surgery, depending on the pathology of the tumor. There are several chemotherapeutic drugs being used to treat lung cancer, including platinum-based agents, taxanes and topoisomerase inhibitors [39, 40]. More recently, gemcitabine, an inhibitor of epidermal growth factor receptor signaling, has been used to treat lung cancer patients [41]. Because chemotherapy decreases the quality of life for patients and is often responsible for serious and sometimes lifethreatening complications, rationally designed tumor specific drugs are especially needed [26]. Natural herbs contain various phenolic compounds, vitamins, carotenoids and flavonoids, and they have been showed various pharmacological effects including antioxidative, antiallergic and anticancer effects [16]. Although anticancer activities of platycodin D have been evaluated including A549 cells, adenocarcinomic human alveolar basal epithelial cells [23, 24], the anticancer effects on H520 cells, a representative NSCLC cell lines of platycodin D did not revealed upon our knowledge. Here, possible antitumor activities of platycodin D were observed using H520 tumor cell-bearing athymic nude mice after continuous oral treatment, once a day for 35 days after confirm the* in vitro* cytotoxicity. The cytotoxicity was tested by MTT assay against H520 cells, and the antitumor, anticachexia and immunomodulatory effects were observed in H520 cell xenograft Balb/c* nu*-*nu* nude mice. In order to investigate whether platycodin D has potent antitumor activities with immunomodulatory effects, 200, 100 and 50 mg/kg of platycodin D was orally administered, once a day for 35 days from 15 days after H520 tumor cell implantation in athymic nude mice, and the changes on body weights, tumor volume and weights, lymphatic organ (spleen and submandibular lymph node), serum IFN-*γ* levels, splenocytes and peritoneal macrophage activities, splenic TNF-*α*, IL-1*β* and IL-10 contents were observed with tumor mass and lymphatic organ histopathology. In addition, changes on the periovarian fat weights and serum IL-6 levels were also detected with the thicknesses of deposited periovarian adipose tissue and their mean diameters to monitor the tumor-related anticachexic effects. In tumor masses, the immunoreactivities of caspase-3 and PARP- apoptotic marks, COX-2, iNOS and TNF-*α* were additionally observed by immunohistochemistry. The results were compared with gemcitabine 160 mg/kg, 7-day intervals, intraperitoneal treated mice, as a reference drug [25, 26].

Platycodin D showed favorable cytotoxic effects on the H520 cells, and also gemcitabine showed favorable cytotoxic effects; IC_50_ of platycodin D and gemcitabine against H520 cells were calculated as 15.86 *μ*g/mL and 456.75 *μ*g/mL (1.74 *μ*M) in this experiment. As results of tumor cell inoculation, marked decreases of spleen and submandibular lymph node weights, serum IFN-*γ*, splenic TNF-*α*, IL-1*β* and IL-10 contents, splenocytes and peritoneal NK cell activities were observed with histopathological atrophic changes of spleen and submandibular lymph nodes. In addition, deceases on the body weights were also demonstrated in tumor-bearing control with increases of serum IL-6 levels, decreases of periovarian fat pad weights, atrophic changes of white adipose tissues, suggesting tumor-related immunosuppress and cachexia. Marked decreases of tumor volumes and weights were demonstrated in gemcitabine 160 mg/kg treated mice with decreases of the tumor cell volumes in the tumor masses were noticed, and also marked increases of the tumor mass caspase-3 and PARP immunoreactivities and decreases of COX-2 immunoreactivities were demonstrated in gemcitabine treated mice as compared with tumor-bearing control. However, gemcitabine treatment aggravated the cancer cachexia (actual body weights, periovarian fat depositions and serum IL-6 levels) and immunosuppress (lymphatic organ weights, serum IFN-*γ* levels, NK cell activities, splenic TNF-*α*, IL-1*β* and IL-10 contents, histopathological atrophic changes of lymphatic organs, iNOS and TNF-*α* immunoreactivities in tumor masses) as compared with tumor-bearing control mice. Platycodin D 200, 100 and 50 mg/kg treated mice showed noticeable immunostimulatory and anticachexia effects with potent antitumor activities as compared with tumor-bearing mice, dose-dependently, in this experiment. These results are indicated that platycodin D has potent antitumor activities through direct cytotoxic effects, increases of apoptosis in tumor cells, immunostimulatory effects, and can be control cancer related cachexia.

Cell growth inhibition assay using MTT is generally used* in vitro* assay to detect possible cytotoxic activities of test materials in chemotherapy, and has been widely used for screening the antitumor activities [42]. In present study, platycodin D showed favorable cytotoxic effects on the H520 cells, IC_50_ of platycodin D against H520 cells were calculated as 15.86 *μ*g/mL in this experiment, similar to those of previous experiments against A549 cells, adenocarcinomic human alveolar basal epithelial cells; platycodin D showed effective cytotoxicity against to A549 cells, IC_50_ of 10–18 *μ*g/mL [23, 24]. Generally, gemcitabine showed 1.54 *μ*M of IC_50_ against H520 [43], and also gemcitabine showed effective cytotoxicity against to H520 cells used in this study as 456.75 *μ*g/mL (1.74 *μ*M) of IC_50_ in this experiment.

Nude mice are congenitally athymic with T lymphocyte related immune deficiency and have served as a useful model for assessing tumor xenograft development [44, 45]. Interestingly, nude mice develop an age-dependent extrathymic T cell maturation, the extent of which is greater in CD8+ cytotoxic than in CD4+ helper T cells [46, 47]. Therefore, it also considered that some immunomodulatory agents can be showed antitumor effect itself or synergic effects with cytotoxic agents mediated by immune stimulated effects, because tumor is directed related with immune deficiency [46, 48]. The antitumor activities in tumor cell xenograft nude mice have been evaluated based on the growth of tumor mass implanted as tumor volumes and weights [29, 49]. In the present study, platycodin D 200, 100 or 50 mg/kg favorably inhibited the tumor growth implanted, dose-dependent, and significant decreases of tumor volumes and weights were detected in all three different dosages of platycodin D administrated mice as compared with tumor-bearing control mice, and also in gemcitabine 160 mg/kg mice as direct evidences that over 50 mg/kg of platycodin D administration induced favorable antitumor effects. In this experiment, platycodin D 100 mg/kg treated mice showed antitumor effects, inhibited the H520 tumor growth as comparable to gemcitabine 160 mg/kg, 7-day intervals, intraperitoneally treatment. These antitumor activities of platycodin D were considered as the results of direct cytotoxicity against H520 cells, induced apoptosis of tumor cells or immunostimulatory effects because platycodin D showed direct favorable cytotoxicity against H520 tumor cells and they showed favorable indirect immunostimulatory effects on lymphatic organs and directly increased various inflammatory mediators and tumor cell apoptosis in tumor masses in this experiment.

T-cell mediated immunosuppress are directly related to cancer occurrences or in cancer patient, marked immune depresses are also observed [12, 13], and recently, stimulation or increases of body immune systems are highlight as a new treatment regimens for cancer therapy [14, 15]. In addition, antioxidants also favorably activated body immune system, and releases of antitumor cytokines and induced antitumor effects [50, 51]. Marked immune deficiencies were also induced in this study after H520 cell implantation, lymphatic organ weights were noticeably reduced in tumor-bearing control mice with marked atrophic changes related to decreases of lymphoid cells in spleen and submandibular lymph nodes at histopathological observations. Although gemcitabine treatment slightly provoked the H520 cell implantation related immunosuppress in a thymic nude mice well corresponded to the previous reports [52, 53], all three different dosages of platycodin D administrated mice showed marked and dose-dependent increase of body immune systems. Once again, the antitumor activities of platycodin D in H520 cell xenograft mice may be mediated, at least partially, by immunostimulatory effects of platycodin D in the present study. Immunomodulatory effects of platycodin D have been revealed through animal experiments [22].

The cytokine TNF-*α*, produced by a variety of cell types, including splenocytes, was found to be associated with critical events leading to T-lineage commitment and differentiation [54]. TNF-*α* can enhance the* in vivo* immune response at doses much lower than those that cause weight loss or tissue toxicity. It enhances proliferation of B and T cells and promotes the generation of cytotoxic T cells. In addition, it enhances IL-2-induced immunoglobulin production and augments IL-2 stimulated natural killer cell activity and proliferation of monocytes [55]. IL-1 is another cytokine released by various cell types such as macrophages, dendritic cells, lymphocytes, endothelial cells, fibroblasts and keratocytes, and two forms of IL-1, IL-1*α* and IL-1*β*. They are both glycoproteins of 17 kDa, and IL-1*β* is secret by cells and IL-1*α* is membrane bounded form. IL-1 is necessary for the successful initiation of some forms of immune response [56]. IL-10 is an immunosuppressive glycoprotein of 19–21 KDa that is secreted by Th2 cells, by some B cells, and by activated macrophages. It is now clear that IL-10 primarily acts on activated macrophages to suppress their secretion of IL-1, IL-12, TNF-*α*, and reactive oxygen radicals [55]. IFN-*γ* is a glycoprotein of 20 to 25 kDa produced by CD8+ T cells, Th1 cells, and NK cells in response to IL-2. It complex effect on B and T cell functions and enhance the NK cell and macrophages activities [55]. We observed that marked decreases of stimulatory cytokines, splenic TNF-*α* and IL-1*β* contents, and blood IFN-*γ* levels, and the inhibitory cytokine levels—splenic IL-10 contents were also decreased as results of decrease of T-lymphocytes after H520 cell xenograft, well corresponded previous studies [12, 13], respectively. However, these splenic and blood cytokine decreases were effectively inhibited by treatment of platycodin D 200, 100 or 50 mg/kg, corresponded to lymphatic organ weights and histopathological inspections, but gemcitabine treatment slightly aggravated the tumor-related immunosuppress in a thymic nude mice as similar as previous reports [52, 53].

As a cancer related immunosuppress, marked functional disorders of various immune cells including NK cells and macrophages have been observed, and activation of these immune cells were highlight as a new treatment regimens for cancer [14, 15]. In this study, marked decreases of NK cell activities were also demonstrated after tumor cell inoculations, but all platycodin D treatment increased the splenic and peritoneal NK cell activities, dose-dependently. Gemcitabine administrated mice showed marked decreases of splenic and peritoneal NK cell activities as compared with tumor-bearing control mice in this experiment.

Apoptosis occurs through two pathways, an extrinsic pathway involving the interaction of death ligands with their respective cell surface receptors and an intrinsic pathway that is initiated by insults that damage the DNA, such as ultraviolet light and chemotherapeutic agents. Both pathways eventually result in mitochondrial damage with release of cytochrome *c* and downstream activation of caspases, such as caspase-3. Activation of other downstream caspases results in cleavage of cellular proteins, such as PARP, cytokeratin 18, and other caspases, which lead to the morphologic and biochemical changes of apoptosis [57, 58]. PARP is a nuclear DNA-binding protein that functions in DNA base excision repair [59]. PARP cleavage results in a decreased enzymatic repair function and contributes to the progression of apoptosis, although PARP cleavage is not absolutely necessary for apoptosis to proceed [60]. Caspase 3, a downstream effector caspase, is responsible for cleavage of several critical nuclear targets in the apoptotic cascade. These include the inhibitor of caspase-activated deoxynuclease, which results in nuclear fragmentation, and PARP, which results in a defective DNA repair function [61]. Detection of the activated caspase 3 and PARP in the tumor mass are indicted apoptosis of tumor cells [62, 63]. In our study, increases of caspase-3 and PARP immunoreactivities were also demonstrated in the tumor masses as gemcitabine and platycodin D administration related tumor cell apoptosis. Platycodin D 200, 100 or 50 mg/kg administrated mice showed significant and dose-dependent increases of the tumor mass caspase-3 and PARP immunolabeled cells as compared with tumor-bearing control mice, as direct evidences that administration of platycodin D favorably potentiated the apoptosis of tumor cells, similar to those of gemcitabine. Platycodin D 100 mg/kg showed similar tumor cell apoptosis as compared to those of gemcitabine 160 mg/kg intraperitoneally treated mice, 7-day intervals. In addition, marked decreases of COX-2, a key enzyme for the synthesis of prostaglandins—chemical mediator of inflammations, is also involved in angiogenesis and progression [64], immunoreactivities were also demonstrated in all test substances administrated mice including gemcitabine and platycodin D 50 mg/kg in the present study, as direct evidences that they inhibited the H520 cell bearing related angiogenesis and progression. Platycodin D 50 mg/kg showed similar inhibitory effects on the tumor cell COX-2 expressions.

Generally, the increases of iNOS activities related to the proinflammatory agents - endotoxin, IL-1*β*, TNF-*α* and interferon-*γ* can be induced shock and over inflammatory responses in the body [65], and over expressions of iNOS also induced tumor neovascularization [66]. However, iNOS produced by activated macrophages can be induced tumor cell apoptosis and related tumor regressions [67]. In the present study, marked and dose-dependent increases of iNOS immunoreactivities were detected in the tumor mass of all platycodin D administered mice. These increases of tumor mass iNOS immunoreactivities are considered as secondary changes from immune stimulatory effects of platycodin D related to NK cell activity. In addition, significant increases of tumor mass TNF-*α*, a representative cytokine involved in tumor necrosis [68] immunoreactivities were also demonstrated in all three different dosages of platycodin D as compared with tumor-bearing control mice, respectively. Gemcitabine did not influence tumor mass iNOS and TNF-*α* immunoreactivities in this experiment.

Cancer cachexia is a paraneoplastic syndrome that worsens the quality of life of patients with a variety of malignant tumors [69, 70]. Numerous studies have suggested that circulating IL-6 secreted from tumor cells plays an important role in cancer-induced cachexia [71–73]. These findings are supported by clinical data that IL-6 might be related to the nutritional status of patients with esophageal cancer [74]. In the present study, marked increases of serum IL-6 levels and cachexia related body weight decrease, reduce and atrophic changes of deposited periovarian fat pads were observed after H520 cell transplantation, but these cachexia related changes were dramatically inhibited by treatment of all three different dosages of platycodin D, dose-dependently, as compared with tumor-bearing control mice, respectively. These are considered as direct evidences that administration of platycodin D, 50 mg/kg, at least, can control the cancer cachexia.

## 5. Conclusion

The results obtained in this study suggest that oral treatment of platycodin D 200, 100, and 50 mg/kg has potent antitumor activities on H520 cells, a representative NSCLC cell line, through direct cytotoxic effects, increases of apoptosis in tumor cells, and immunostimulatory effects and can control cancer related cachexia. Platycodin D 100 mg/kg showed potent antitumor effects as comparable with gemcitabine 160 mg/kg intraperitoneally treated mice, 7-day intervals. Gemcitabine aggravated the cancer-related immune suppressions and cachexia in this experiment.

## Figures and Tables

**Table 1:** Primary antisera and detection kits used in this study.

Antisera or detection kits	Code	Source	Dilution
Primary antisera^*^			
Anticleaved caspase-3 (Asp175) polyclonal antibody	9661	Cell Signaling Technology Inc, MA, USA	1 : 400
Anticleaved PARP (Asp214) rat specific antibody	9545	Cell Signaling Technology Inc, MA, USA	1 : 100
Antitumor necrosis factor-*α* (4E1) antibody	sc-130349	Santa Cruz Biotechnology, CA, USA	1 : 200
Anticyclooxygenase (murine) polyclonal antibody	160126	Cayman Chemical., MI, USA	1 : 200
Anti-nitric oxide synthase2 (N-20) polyclonal antibody	sc-651	Santa Cruz Biotechnology, CA, USA	1 : 100
Detection kits			
Vectastain Elite ABC Kit	PK-6200	Vector Lab. Inc., CA, USA	1 : 50
Peroxidae substrate kit	SK-4100	Vector Lab. Inc., CA, USA	1 : 50

^*^All antisera were diluted by phosphate buffered saline (pH 7.2).

**Table 2:** Tumor mass and organ weights in H520 tumor cell xenograft mice.

Organs	Groups					
	Intact control	TB control	Gemcitabine	Platycodin D		
				200 mg/kg	100 mg/kg	50 mg/kg
Absolute weight (g)						
Tumor mass		6.887 ± 0.991	3.674 ± 0.450^c^	2.105 ± 0.366^c^	3.559 ± 0.679^c^	4.235 ± 0.528^c^
Spleen	0.104 ± 0.020	0.040 ± 0.005^d^	0.034 ± 0.009^df^	0.071 ± 0.009^de^	0.059 ± 0.007^de^	0.051 ± 0.008^de^
Submandibular lymph node	0.013 ± 0.002	0.002 ± 0.001^a^	0.002 ± 0.001^a^	0.010 ± 0.002^ac^	0.007 ± 0.001^ac^	0.005 ± 0.001^ac^
Periovarian fat pad	0.167 ± 0.050	0.017 ± 0.007^d^	0.009 ± 0.002^de^	0.074 ± 0.021^de^	0.059 ± 0.010^de^	0.049 ± 0.013^de^
Relative weights (% of body weight)						
Tumor mass		37.486 ± 7.219	23.167 ± 4.119^e^	10.065 ± 1.519^e^	17.643 ± 3.356^e^	23.103 ± 2.919^e^
Spleen	0.449 ± 0.110	0.216 ± 0.029^d^	0.216 ± 0.067^d^	0.340 ± 0.052^e^	0.294 ± 0.047^de^	0.277 ± 0.049^df^
Submandibular lymph node	0.054 ± 0.008	0.012 ± 0.005^a^	0.012 ± 0.007^a^	0.046 ± 0.007^bc^	0.035 ± 0.006^ac^	0.027 ± 0.007^ac^
Periovarian fat pad	0.725 ± 0.249	0.091 ± 0.033^d^	0.057 ± 0.012^df^	0.353 ± 0.100^de^	0.295 ± 0.052^de^	0.264 ± 0.065^de^

Values are expressed mean ± S.D. of eight mice.

TB: tumor bearing.

Gemcitabine was intraperitoneally administered at 160 mg/kg, 7-day intervals.

Platycodin D was orally administered, once a day.

^a^ *P* < 0.01 and ^b^ *P* < 0.05 as compared with intact control by LSD test.

^c^ *P* < 0.01 as compared with TB control by LSD test.

^d^ *P* < 0.01 as compared with intact control by MW test.

^e^ *P* < 0.01 and ^f^ *P* < 0.05 as compared with TB control by MW test.

**Table 3:** Changes on the splenic cytokine contents in H520 tumor cell xenograft mice.

Groups	Tumor necrosis factor-*α*	Interleukin-1*β*	Interleukin-10
Controls			
Intact	98.63 ± 10.54	33.69 ± 5.72	88.98 ± 18.71
TB	42.22 ± 10.76^a^	9.45 ± 2.22^d^	37.19 ± 7.35^a^
Reference			
Gemcitabine	29.47 ± 10.28^ac^	6.86 ± 1.49^df^	27.61 ± 7.10^a^
Platycodin D			
200 mg/kg	72.17 ± 10.23^ac^	21.52 ± 5.77^de^	69.70 ± 11.58^ab^
100 mg/kg	82.95 ± 6.13^ac^	17.25 ± 2.31^de^	58.32 ± 11.10^ab^
50 mg/kg	57.50 ± 7.92^ac^	12.32 ± 2.19^de^	50.72 ± 12.56^ac^

Values are expressed mean ± S.D. of eight mice, pg/mg protein.

TB: tumor-bearing.

Gemcitabine was intraperitoneally administered at 160 mg/kg, 7-day intervals.

Platycodin D was orally administered, once a day.

^a^ *P* < 0.01 as compared with intact control by LSD test.

^b^ *P* < 0.01 and ^c^ *P* < 0.05 as compared with TB control by LSD test.

^d^ *P* < 0.01 as compared with intact control by MW test.

^e^ *P* < 0.01 and ^f^ *P* < 0.05 as compared with TB control by MW test.

**Table 4:** Changes on the tumor mass histomorphometry in H520 tumor cell xenograft mice.

Index	Groups				
	TB control	Gemcitabine	Platycodin D		
			200 mg/kg	100 mg/kg	50 mg/kg
Tumor cell volume (%/mm^2^)	80.25 ± 7.17	49.97 ± 7.83^a^	28.80 ± 6.86^a^	48.96 ± 6.97^a^	63.18 ± 7.79^a^
Immunoreactive cell percentages (%/tumor cells)					
Caspase-3	10.38 ± 2.83	55.13 ± 11.78^a^	81.13 ± 11.69^a^	56.63 ± 9.33^a^	35.50 ± 10.07^a^
Cleaved poly(ADP-ribose) polymerase	17.25 ± 3.37	58.00 ± 12.54^a^	80.38 ± 12.53^a^	61.13 ± 14.44^a^	44.75 ± 11.84^a^
Cyclooxygenase-2	60.88 ± 16.28	41.88 ± 11.08^a^	16.13 ± 5.19^a^	38.38 ± 12.74^a^	47.63 ± 10.23^b^
Inducible nitric oxide synthases	11.25 ± 3.69	11.50 ± 3.34	59.50 ± 13.51^c^	46.00 ± 8.59^c^	25.63 ± 7.85^c^
Tumor necrosis factor-*α*	9.38 ± 1.69	8.50 ± 2.45	59.38 ± 12.22^c^	44.63 ± 10.64^c^	30.75 ± 12.79^c^

Values are expressed mean ± S.D. of eight mice.

TB: tumor-bearing.

Gemcitabine was intraperitoneally administered at 160 mg/kg, 7-day intervals.

Platycodin D was orally administered, once a day.

^a^ *P* < 0.01 and ^b^ *P* < 0.05 as compared with TB control by LSD test.

^c^ *P* < 0.01 as compared with TB control by MW test.

**Table 5:** Changes on the spleen, submandibular lymph node, and periovarian fat fad histomorphometry in H520 tumor cell xenograft mice.

Organs	Groups					
	Intact control	TB control	Gemcitabine	Platycodin D		
				200 mg/kg	100 mg/kg	50 mg/kg
Spleen						
Total thickness (mm)	1.75 ± 0.24	1.08 ± 0.17^a^	1.02 ± 0.18^a^	1.50 ± 0.14^ab^	1.39 ± 0.14^ab^	1.29 ± 0.15^ac^
White pulp numbers (/mm^2^)	15.75 ± 2.92	5.50 ± 1.41^a^	5.13 ± 1.25^a^	13.13 ± 1.46^ab^	11.00 ± 2.14^ab^	8.13 ± 1.13^ab^
White pulp diameters (*μ*m)	489.11 ± 110.18	213.69 ± 30.71^d^	216.96 ± 40.09^d^	361.61 ± 50.97^ef^	309.88 ± 73.63^df^	281.75 ± 64.50^de^
Submandibular lymph node						
Total thickness (*μ*m)	993.00 ± 147.40	529.98 ± 72.52^a^	557.65 ± 74.26^a^	804.88 ± 112.71^ab^	753.23 ± 109.45^ab^	701.97 ± 97.42^ab^
Cortex follicle numbers (/mm^2^)	17.13 ± 1.55	6.63 ± 1.06^a^	6.25 ± 1.83^a^	13.50 ± 2.88^ab^	11.88 ± 2.42^ab^	9.88 ± 1.96^ab^
Cortex thickness (*μ*m)	502.88 ± 101.13	245.84 ± 40.07^d^	239.39 ± 39.83^d^	408.00 ± 52.31^ef^	360.78 ± 50.06^df^	299.68 ± 28.60^dg^
Periovarian fat pad						
Total thickness (mm)	1.84 ± 0.25	0.42 ± 0.06^d^	0.33 ± 0.07^dg^	1.24 ± 0.23^df^	0.97 ± 0.11^df^	0.75 ± 0.19^df^
White adipocyte diameters (*μ*m)	50.23 ± 5.93	16.10 ± 2.51^d^	10.70 ± 2.20^dg^	35.59 ± 5.95^df^	28.07 ± 4.03^df^	23.16 ± 4.15^df^

Values are expressed mean ± S.D. of eight mice.

TB = tumor bearing.

Gemcitabine was intraperitoneally administered at 160 mg/kg, 7-day intervals.

Platycodin D was orally administered, once a day.

^a^ *P* < 0.01 as compared with intact control by LSD test.

^b^ *P* < 0.01 and ^c^ *P* < 0.05 as compared with TB control by LSD test.

^d^ *P* < 0.01 and ^e^ *P* < 0.05 as compared with intact control by MW test.

^f^ *P* < 0.01 and ^g^ *P* < 0.05 as compared with TB control by MW test.
